# Tetra­aqua­bis(pyridine-3-sulfonato-κ*N*)nickel(II)

**DOI:** 10.1107/S1600536808010076

**Published:** 2008-04-16

**Authors:** Bing-Yu Zhang, Jing-Jing Nie, Duan-Jun Xu

**Affiliations:** a Department of Chemistry, Zhejiang University, Hangzhou 310027, People’s Republic of China

## Abstract

In the mol­ecule of the title compound, [Ni(C_5_H_4_NO_3_S)_2_(H_2_O)_4_], the Ni^II^ cation is located on an inversion center and is coordinated by four water mol­ecules and two pyridine-3-sulfonate anions with an NiN_2_O_4_ distorted octa­hedral geometry. The face-to-face separation of 3.561 (5) Å between parallel pyridine rings indicates the existence of weak π–π stacking between the pyridine rings. The structure also contains inter­molecular O—H⋯O hydrogen bonding and weak C—H⋯O hydrogen bonding.

## Related literature

For general background, see: Deisenhofer & Michel (1989 ▶); Su & Xu (2004 ▶); Liu *et al.* (2004 ▶); Li *et al.* (2005 ▶). For a related structure, see: Walsh & Hathaway (1980 ▶). For related literature, see: Cotton & Wilkinson (1972 ▶).
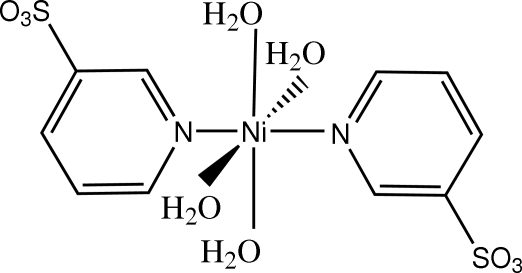


## Experimental

### 

#### Crystal data

[Ni(C_5_H_4_NO_3_S)_2_(H_2_O)_4_]
*M* *_r_* = 447.08
Monoclinic, 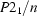

*a* = 7.5399 (8) Å
*b* = 12.6939 (15) Å
*c* = 8.7810 (8) Å
β = 97.419 (12)°
*V* = 833.40 (15) Å^3^
*Z* = 2
Mo *K*α radiation
μ = 1.47 mm^−1^
*T* = 295 (2) K
0.32 × 0.22 × 0.20 mm

#### Data collection

Rigaku R-AXIS RAPID IP diffractometer
Absorption correction: multi-scan (*ABSCOR*; Higashi, 1995 ▶) *T*_min_ = 0.660, *T*_max_ = 0.745
8877 measured reflections
1524 independent reflections
1449 reflections with *I* > 2σ(*I*)
*R*_int_ = 0.019

#### Refinement

*R*[*F* ^2^ > 2σ(*F* ^2^)] = 0.024
*wR*(*F* ^2^) = 0.065
*S* = 1.06
1524 reflections
115 parameters
H-atom parameters constrained
Δρ_max_ = 0.29 e Å^−3^
Δρ_min_ = −0.37 e Å^−3^

### 

Data collection: *PROCESS-AUTO* (Rigaku, 1998 ▶); cell refinement: *PROCESS-AUTO*; data reduction: *CrystalStructure* (Rigaku/MSC, 2002 ▶); program(s) used to solve structure: *SIR92* (Altomare *et al.*, 1993 ▶); program(s) used to refine structure: *SHELXL97* (Sheldrick, 2008 ▶); molecular graphics: *ORTEP-3 for Windows* (Farrugia, 1997 ▶); software used to prepare material for publication: *WinGX* (Farrugia, 1999 ▶).

## Supplementary Material

Crystal structure: contains datablocks I, global. DOI: 10.1107/S1600536808010076/om2227sup1.cif

Structure factors: contains datablocks I. DOI: 10.1107/S1600536808010076/om2227Isup2.hkl

Additional supplementary materials:  crystallographic information; 3D view; checkCIF report

## Figures and Tables

**Table 1:** Selected bond lengths (Å)

Ni—O1	2.0828 (14)
Ni—O2	2.0739 (14)
Ni—N1	2.1026 (16)

**Table 2:** Hydrogen-bond geometry (Å, °)

*D*—H⋯*A*	*D*—H	H⋯*A*	*D*⋯*A*	*D*—H⋯*A*
O1—H1*A*⋯O3^i^	0.81	2.39	3.162 (3)	158
O1—H1*A*⋯O4^i^	0.81	2.44	3.119 (2)	142
O1—H1*B*⋯O4^ii^	0.89	1.83	2.722 (2)	177
O2—H2*A*⋯O3^iii^	0.84	1.97	2.787 (2)	163
O2—H2*B*⋯O5^iv^	0.86	1.89	2.748 (2)	174
C1—H1⋯O4^i^	0.93	2.34	3.212 (3)	155

Symmetry codes: (i) 
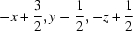
; (ii) 
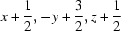
; (iii) 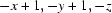; (iv) 
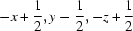
.

**Fig. 1.**
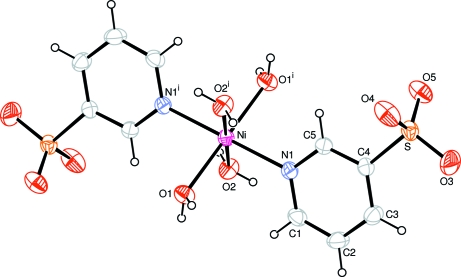
The molecular structure of the title compound with 40% probability displacement (arbitrary spheres for H atoms) [symmetry codes: (i)1 - x,1 - y,1 - z].

**Fig. 2.**
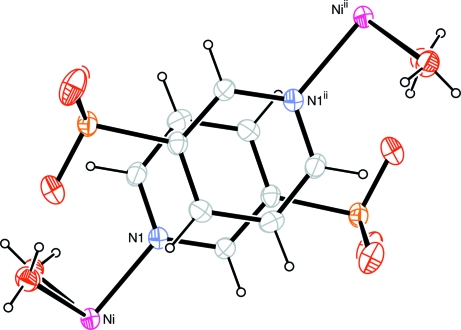
A diagram showing π-π stacking between pyridine rings [symmetry code: (ii) 1 - x,1 - y,-z].

## supplementary crystallographic information

### Comment

As π-π stacking between aromatic rings plays an important role in electron transfer process in some biological system (Deisenhofer & Michel, 1989), π-π stacking has attracted our much attention in past years (Su & Xu, 2004; Liu *et al.*, 2004; Li *et al.*, 2005). In order to investigate the influence of substituents on aromatic stacking, the title pyridine-sulfate complex has recently prepared and its crystal structure is reported here.

The molecular structure of the title compound is shown in Fig. 1. The Ni^II^ cation is located in an inversion center and coordinated by four water molecules and two pyridine-3-sulfonate anions with a NiN_2_O_4_ distorted octahedral geometry (Table 1), similar to the analogue of Zn^II^ (Walsh & Hathaway, 1980). Partially overlapped arrangement is observed between parallel pyridine rings (Fig. 2). The face-to-face separation of 3.561 (5) Å between parallel pyridine rings is shorter than the van der Waals thickness of an aromatic ring (3.70 Å; Cotton & Wilkinson, 1972), and indicates the existence of weak π–π stacking between the pyridine rings.

The intermolecular O—H···O hydrogen bonding and weak C—H···O hydrogen bonding (Table 2) help to stabilize the crystal structure.

### Experimental

Pyridine-3-sulfonic acid (0.159 g, 1 mmol), Na_2_CO_3_ (0.053 g, 0.5 mmol), NiCl_2_.6H_2_O (0.238 g, 1 mmol) were dissolved in a mixture solution of water (8 ml) and ethanol (2 ml). The solution was placed in a 15 ml Teflon-lined stainless steel autoclave under autogenous pressure at 398 K for 75 h and filtered after cooling to room temperature. The blue single crystals of the title compound were obtained from the filtrate after 3 months.

### Refinement

Water H atoms were located in a difference Fourier map and refined as riding in as-found relative positions with *U*_iso_(H) = 1.5*U*_eq_(O). Aromatic H atoms were placed in calculated positions with C—H = 0.93 Å and refined in riding mode with *U*_iso_(H) = 1.2*U*_eq_(C).

### Crystal data

**Table:**

[Ni(C_5_H_4_NO_3_S)_2_(H_2_O)_4_]	*F*_000_ = 460
*M**_r_* = 447.08	*D*_x_ = 1.782 Mg m^−^^3^
Monoclinic, *P*2_1_/*n*	Mo *K*α radiation λ = 0.71073 Å
Hall symbol: -P 2yn	Cell parameters from 5256 reflections
*a* = 7.5399 (8) Å	θ = 2.8–24.0º
*b* = 12.6939 (15) Å	µ = 1.47 mm^−^^1^
*c* = 8.7810 (8) Å	*T* = 295 (2) K
β = 97.419 (12)º	Prism, blue
*V* = 833.40 (15) Å^3^	0.32 × 0.22 × 0.20 mm
*Z* = 2	

### Data collection

**Table:**

Rigaku R-AXIS RAPID IP diffractometer	1524 independent reflections
Radiation source: fine-focus sealed tube	1449 reflections with *I* > 2σ(*I*)
Monochromator: graphite	*R*_int_ = 0.019
Detector resolution: 10.0 pixels mm^-1^	θ_max_ = 25.4º
*T* = 295(2) K	θ_min_ = 2.8º
ω scans	*h* = −9→9
Absorption correction: multi-scan(ABSCOR; Higashi, 1995)	*k* = −15→15
*T*_min_ = 0.660, *T*_max_ = 0.745	*l* = −9→10
8877 measured reflections	

### Refinement

**Table:**

Refinement on *F*^2^	Secondary atom site location: difference Fourier map
Least-squares matrix: full	Hydrogen site location: inferred from neighbouring sites
*R*[*F*^2^ > 2σ(*F*^2^)] = 0.024	H-atom parameters constrained
*wR*(*F*^2^) = 0.065	*w* = 1/[σ^2^(*F*_o_^2^) + (0.0323*P*)^2^ + 0.5265*P*] where *P* = (*F*_o_^2^ + 2*F*_c_^2^)/3
*S* = 1.06	(Δ/σ)_max_ < 0.001
1524 reflections	Δρ_max_ = 0.29 e Å^−^^3^
115 parameters	Δρ_min_ = −0.37 e Å^−^^3^
Primary atom site location: structure-invariant direct methods	Extinction correction: none

### Special details

**Table:**

Geometry. All e.s.d.'s (except the e.s.d. in the dihedral angle between two l.s. planes) are estimated using the full covariance matrix. The cell e.s.d.'s are taken into account individually in the estimation of e.s.d.'s in distances, angles and torsion angles; correlations between e.s.d.'s in cell parameters are only used when they are defined by crystal symmetry. An approximate (isotropic) treatment of cell e.s.d.'s is used for estimating e.s.d.'s involving l.s. planes.
Refinement. Refinement of *F*^2^ against ALL reflections. The weighted *R*-factor *wR* and goodness of fit *S* are based on *F*^2^, conventional *R*-factors *R* are based on *F*, with *F* set to zero for negative *F*^2^. The threshold expression of *F*^2^ > σ(*F*^2^) is used only for calculating *R*-factors(gt) *etc*. and is not relevant to the choice of reflections for refinement. *R*-factors based on *F*^2^ are statistically about twice as large as those based on *F*, and *R*- factors based on ALL data will be even larger.

### Fractional atomic coordinates and isotropic or equivalent isotropic displacement parameters (Å^2^)

**Table:**

	*x*	*y*	*z*	*U*_iso_*/*U*_eq_	
Ni	0.5000	0.5000	0.5000	0.02883 (13)	
S	0.45204 (7)	0.75905 (4)	−0.03581 (6)	0.03407 (15)	
N1	0.5850 (2)	0.54221 (13)	0.28948 (18)	0.0307 (3)	
O1	0.76142 (19)	0.48676 (11)	0.60868 (18)	0.0388 (3)	
H1B	0.8326	0.5397	0.5900	0.058*	
H1A	0.8182	0.4333	0.5998	0.058*	
O2	0.50231 (19)	0.34035 (11)	0.45010 (17)	0.0411 (3)	
H2A	0.5076	0.3156	0.3615	0.062*	
H2B	0.4194	0.3021	0.4815	0.062*	
O3	0.5370 (3)	0.76975 (16)	−0.1732 (2)	0.0687 (6)	
O4	0.4828 (2)	0.85025 (12)	0.0624 (2)	0.0554 (5)	
O5	0.2658 (2)	0.73019 (12)	−0.06337 (19)	0.0481 (4)	
C1	0.7217 (3)	0.49242 (15)	0.2368 (3)	0.0370 (5)	
H1	0.7768	0.4372	0.2943	0.044*	
C2	0.7839 (3)	0.51907 (18)	0.1020 (3)	0.0452 (5)	
H2	0.8783	0.4819	0.0693	0.054*	
C3	0.7053 (3)	0.60162 (17)	0.0150 (3)	0.0405 (5)	
H3	0.7461	0.6218	−0.0762	0.049*	
C4	0.5641 (2)	0.65298 (14)	0.0684 (2)	0.0298 (4)	
C5	0.5074 (3)	0.62143 (15)	0.2044 (2)	0.0320 (4)	
H5	0.4116	0.6565	0.2384	0.038*	

### Atomic displacement parameters (Å^2^)

**Table:**

	*U*^11^	*U*^22^	*U*^33^	*U*^12^	*U*^13^	*U*^23^
Ni	0.0309 (2)	0.0273 (2)	0.0284 (2)	0.00105 (13)	0.00426 (14)	0.00418 (13)
S	0.0419 (3)	0.0278 (3)	0.0317 (3)	0.0027 (2)	0.0018 (2)	0.00399 (19)
N1	0.0329 (8)	0.0290 (8)	0.0302 (8)	0.0016 (7)	0.0037 (6)	0.0034 (7)
O1	0.0336 (7)	0.0355 (8)	0.0467 (9)	0.0018 (6)	0.0031 (6)	0.0056 (6)
O2	0.0512 (9)	0.0328 (8)	0.0409 (8)	−0.0017 (6)	0.0122 (7)	−0.0002 (6)
O3	0.0861 (14)	0.0758 (13)	0.0491 (11)	0.0261 (11)	0.0269 (10)	0.0305 (9)
O4	0.0591 (10)	0.0297 (8)	0.0707 (11)	0.0048 (7)	−0.0174 (8)	−0.0095 (8)
O5	0.0441 (9)	0.0358 (8)	0.0600 (10)	0.0039 (7)	−0.0103 (7)	−0.0022 (7)
C1	0.0360 (11)	0.0328 (11)	0.0417 (12)	0.0064 (8)	0.0034 (9)	0.0061 (8)
C2	0.0417 (12)	0.0431 (12)	0.0542 (14)	0.0126 (10)	0.0188 (10)	0.0065 (10)
C3	0.0455 (12)	0.0393 (11)	0.0395 (12)	0.0036 (9)	0.0154 (9)	0.0055 (9)
C4	0.0337 (9)	0.0255 (9)	0.0295 (10)	−0.0002 (7)	0.0016 (8)	0.0005 (7)
C5	0.0333 (9)	0.0314 (10)	0.0313 (10)	0.0041 (8)	0.0047 (8)	0.0004 (8)

### Geometric parameters (Å, °)

**Table:**

Ni—O1^i^	2.0828 (14)	O1—H1B	0.8886
Ni—O1	2.0828 (14)	O1—H1A	0.8115
Ni—O2^i^	2.0739 (14)	O2—H2A	0.8450
Ni—O2	2.0739 (14)	O2—H2B	0.8642
Ni—N1^i^	2.1026 (16)	C1—C2	1.370 (3)
Ni—N1	2.1026 (16)	C1—H1	0.9300
S—O5	1.4412 (16)	C2—C3	1.384 (3)
S—O3	1.4438 (18)	C2—H2	0.9300
S—O4	1.4445 (16)	C3—C4	1.381 (3)
S—C4	1.7788 (19)	C3—H3	0.9300
N1—C1	1.341 (3)	C4—C5	1.379 (3)
N1—C5	1.341 (2)	C5—H5	0.9300
			
O2^i^—Ni—O2	180.0	C5—N1—Ni	121.36 (13)
O2^i^—Ni—O1^i^	89.12 (6)	Ni—O1—H1B	114.4
O2—Ni—O1^i^	90.88 (6)	Ni—O1—H1A	120.2
O2^i^—Ni—O1	90.88 (6)	H1B—O1—H1A	106.0
O2—Ni—O1	89.12 (6)	Ni—O2—H2A	124.1
O1^i^—Ni—O1	180.0	Ni—O2—H2B	117.1
O2^i^—Ni—N1^i^	92.95 (6)	H2A—O2—H2B	101.9
O2—Ni—N1^i^	87.05 (6)	N1—C1—C2	123.07 (19)
O1^i^—Ni—N1^i^	92.61 (6)	N1—C1—H1	118.5
O1—Ni—N1^i^	87.39 (6)	C2—C1—H1	118.5
O2^i^—Ni—N1	87.05 (6)	C1—C2—C3	119.6 (2)
O2—Ni—N1	92.95 (6)	C1—C2—H2	120.2
O1^i^—Ni—N1	87.39 (6)	C3—C2—H2	120.2
O1—Ni—N1	92.61 (6)	C4—C3—C2	117.6 (2)
N1^i^—Ni—N1	180.0	C4—C3—H3	121.2
O5—S—O3	114.30 (12)	C2—C3—H3	121.2
O5—S—O4	112.45 (10)	C5—C4—C3	119.71 (18)
O3—S—O4	111.67 (12)	C5—C4—S	119.06 (14)
O5—S—C4	106.36 (9)	C3—C4—S	121.23 (15)
O3—S—C4	105.58 (10)	N1—C5—C4	122.60 (18)
O4—S—C4	105.69 (9)	N1—C5—H5	118.7
C1—N1—C5	117.40 (17)	C4—C5—H5	118.7
C1—N1—Ni	121.22 (13)		

Symmetry codes: (i) −*x*+1, −*y*+1, −*z*+1.

### Hydrogen-bond geometry (Å, °)

**Table:**

*D*—H···*A*	*D*—H	H···*A*	*D*···*A*	*D*—H···*A*
O1—H1A···O3^ii^	0.81	2.39	3.162 (3)	158
O1—H1A···O4^ii^	0.81	2.44	3.119 (2)	142
O1—H1B···O4^iii^	0.89	1.83	2.722 (2)	177
O2—H2A···O3^iv^	0.84	1.97	2.787 (2)	163
O2—H2B···O5^v^	0.86	1.89	2.748 (2)	174
C1—H1···O4^ii^	0.93	2.34	3.212 (3)	155

Symmetry codes: (ii) −*x*+3/2, *y*−1/2, −*z*+1/2; (iii) *x*+1/2, −*y*+3/2, *z*+1/2; (iv) −*x*+1, −*y*+1, −*z*; (v) −*x*+1/2, *y*−1/2, −*z*+1/2.
